# Boundary Layer Effect on Behavior of Discrete Models

**DOI:** 10.3390/ma10020157

**Published:** 2017-02-10

**Authors:** Jan Eliáš

**Affiliations:** Brno University of Technology, Faculty of Civil Engineering, Veveří 331/95, Brno 60200, Czech Republic; elias.j@fce.vutbr.cz; Tel.: +420-541-147-132

**Keywords:** boundary layer, wall effect, discrete model, elasticity, fracture, concrete

## Abstract

The paper studies systems of rigid bodies with randomly generated geometry interconnected by normal and tangential bonds. The stiffness of these bonds determines the macroscopic elastic modulus while the macroscopic Poisson’s ratio of the system is determined solely by the normal/tangential stiffness ratio. Discrete models with no directional bias have the same probability of element orientation for any direction and therefore the same mechanical properties in a statistical sense at any point and direction. However, the layers of elements in the vicinity of the boundary exhibit biased orientation, preferring elements parallel with the boundary. As a consequence, when strain occurs in this direction, the boundary layer becomes stiffer than the interior for the normal/tangential stiffness ratio larger than one, and vice versa. Nonlinear constitutive laws are typically such that the straining of an element in shear results in higher strength and ductility than straining in tension. Since the boundary layer tends, due to the bias in the elemental orientation, to involve more tension than shear at the contacts, it also becomes weaker and less ductile. The paper documents these observations and compares them to the results of theoretical analysis.

## 1. Introduction

The presence of a boundary is inevitable for any solid. The material in the vicinity of the boundary often has different material properties to that lying further from the boundary due to various effects. In concrete, one of the main effects is that the boundary layer typically contains a lower amount of larger mineral aggregates and more small aggregates and mortar compared to the interior material [1]. The boundary layer thickness is determined by the sieve curve of the material and is independent of the size of the specimen/member. The presence of the boundary layer may affect the elastic and inelastic mechanical behavior of concrete members.

In the numerical analysis of concrete members using continuous homogeneous models, the boundary layer is typically not represented. However, some approaches lead to the creation of a boundary layer that is different from the interior (nonlocal models [2], for example) or a boundary layer is directly created on purpose [3]. In the case of mesoscale models, the different distribution of mineral aggregates in the boundary layer can be directly imposed when creating the model.

The paper focuses on a specific type of discrete mesoscale model with random geometry where every model node represents one larger aggregate and its surroundings. This type of model is often called a particle model [4,5]. Its advantage, compared to the classical lattice models of concrete [6,7,8] with material structure projected onto the independent lattice structure, is the great reduction in computational time that it provides. Specifically, the focus in this paper is placed on models with geometry generated via Voronoi or similar tessellation [9,10,11].

It will be demonstrated that the boundary layer is inevitable in this type of model, and affects its mechanical behavior. For a positive Poisson’s ratio, the boundary layer becomes more compliant than the bulk material, while, for a negative Poisson’s ratio, it becomes stiffer. In the nonlinear regime, the boundary layer is weaker and less ductile than the interior material. All of these phenomena are consequences of geometrical bias in the boundary layer. While the elements inside the specimen are oriented with the same probability in any direction, the boundary layer has more elements oriented along the boundary.

When the discrete model is understood to be only some kind of discretization technique, the presence of a boundary layer with a thickness that is related to the discretization density is inconvenient. Recently, an iterative technique capable of removing both elastic stress fluctuations and the elastic effects of the boundary layer with no limits in Poisson’s ratio has been developed [12,13]. The disadvantage of this approach is the loss of splitting forces under compression. However, the boundary layer might still have an effect in the nonlinear regime, depending on the applied constitutive law. Another approach with similar consequences uses a constitutive law based on the volumetric-deviatoric split of a strain tensor [14].

Viewing the discrete particle model not as a discretization technique but as a meso-level model mimicking real material structure, the aforementioned boundary layer might also be viewed as realistic. However, the underlying origin of the boundary layer in the numerical model is completely different from a real heterogeneous solid. Because there are no experiments known to the author that evaluate the boundary layer effect, it is not possible to determine its appropriateness in the model.

The author was confronted with boundary layer effects for the first time in [15], where the elastic tensorial stress in the boundary layer layer clearly deviated from the theoretical behavior and exhibited strong dependence on Poisson’s ratio. Another influence of the boundary layer in the elastic regime was noted in [16]. The macroscopic elastic modulus and Poisson’s ratio deviated for decreasing discretization density. This was caused by the increasing fraction of the boundary layer in the specimen volume. Finally, in the nonlinear regime, the adaptive technique [16] showed sensitivity to the construction of the model geometry in the boundary region. All of these effects are explained by the findings presented in this paper.

## 2. Model

The studied model is a discrete system of rigid bodies connected at common facets by linear or nonlinear contacts. The constitutive equations are taken from [17,18] and simplified. A full description of both linear and nonlinear constitutive equations, model kinematics and the creation of the model geometry is provided in [16]. The interested reader is kindly asked to find all additional model-related information there, as, for sake of simplicity, only the most necessary information is included in this paper.

The kinematics of the bodies is dictated by rigid body motion assuming small rotations, and is thus often called a rigid-body–spring network [19,20]. Each body has three degrees of freedom (DOFs) in two dimensions (2 × translation *u*, 1 × rotation *φ*), and six in three dimensions (3 × translation *u*, 3 × rotation *φ*). Both the 2D and 3D versions of the model are used in this paper.

**Figure 1:**
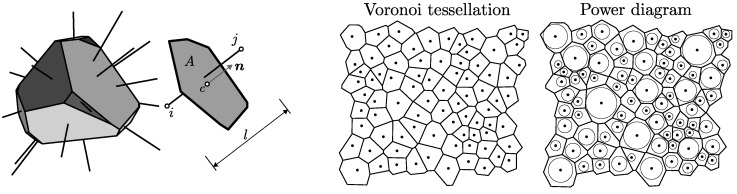
left: one rigid body and one contact facet connecting nodes *i* and *j* with normal n, length *l* and area *A*; right: Voronoi tessellation and Power diagram for the same set of nuclei.

One rigid body and one facet are shown in Figure 1. The orientation of the facet is described by its *normal* direction, n; the length of the contact is denoted $l=∥x_{i}-x_{j}∥$, its area is *A* and centroid is c.

**Figure 2:**
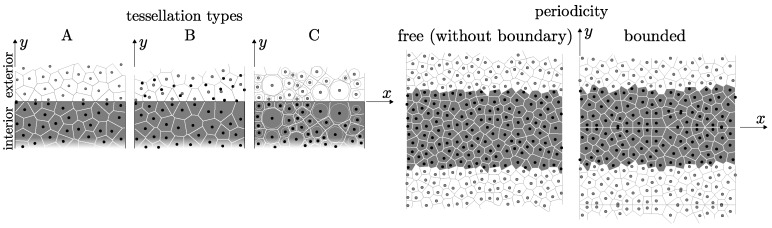
left: different tessellation types in the boundary region; right: 2D sketch of periodic structure with and without a boundary.

Three algorithms are used for geometry construction. The first two types are controlled by the parameter $l_{\min}$, while the third type needs a sieve curve of the mineral aggregates on input. The differences between the tessellation types are schematically demonstrated in 2D in Figure 2 left.

- In the first type (denoted A), the specimen domain is randomly filled with nuclei that are not closer to each other than $l_{\min}$. The filling is carried out sequentially, one nucleus at a time, and stops after a large number of trial nuclei have been rejected due to violation of the minimum distance. Then, Voronoi tessellation is performed to divide the domain into bodies. The rigid body associated with nucleus *i* encompasses all the points x that are closer to *i* than to any other nucleus. Voronoi faces, here referred to as facets, represents the bonds between two bodies. The boundary region requires special treatment. According to Bolander et al. [20], the nuclei are mirrored across the boundary before performing the Voronoi tessellation. The tessellation then creates the boundaries of the rigid bodies exactly at the boundaries of the specimen.
- The second tessellation type (denoted B) differs only slightly from the first. The nuclei are randomly placed into a larger volume than needed. After the random placing is complete, nuclei outside the true specimen domain are removed. The tessellation and the treatment of the boundary region both proceed exactly as in case A. The only difference between the A and B tessellation is in the random placing of the nuclei in the boundary region. In case A, the presence of the boundary already affects the placing process; while, in case B, the boundary is effective only during the tessellation.
- Finally, tessellation type C accounts for different aggregate sizes. Spherical aggregates of different radii, *r*, are generated based on the sieve curve. The Fuller curve with 75% of aggregate volume with radii within the range $l_{\min}-0.2l_{\min}$ is used. The aggregates are sequentially randomly placed into the domain, starting with the largest ones. No overlapping of the boundary is allowed, and the minimal mutual distance between two spherical centers is 1.1 × the sum of their radii. The power diagram [21] is used instead of the Voronoi tessellation. The power diagram, which is a weighted version of Voronoi tessellation, is based on the power of the point to the sphere defined as $d^{2}-r^{2}$, where *d* is the distance of the point from the spherical center and *r* is the sphere radius. The body of the power diagram associated with sphere *i* is a set of all points with lower power to sphere *i* than to any other sphere. The power diagram is computed with the help of [22].

The last modification of the tessellation procedure is periodic repetition of the model structure. The periodicity allows for complete removal of the boundary. Though it can be generally used with tessellation types A or C, it is employed here only in connection with A. During random placement of nuclei, every nucleus is periodically repeated twice in the *y*- and *z*-directions. The tessellation is then performed on a periodic structure that is 3 × larger in both the *y*- and *z*-directions. When solution of the mechanical system is carried out, periodic images of nuclei have dependent DOFs and only one periodic image of each contact contributes to the strain energy and is therefore included in the stiffness matrix. This approach completely removes boundaries and creates ideal directionally unbiased geometry. When boundaries are present in the periodic structure, the nuclei in the central part of the prismatic specimen are mirrored across the xy and xz plane. After tessellation, the planes xy and xz behave in the same way as a specimen boundary with directionally biased geometry in its vicinity. An example of the periodic boundary *free* and periodic *bounded* structures in 2D is shown in Figure 2.

All of the three tessellation types have two fundamental features used later in the theoretical derivations: (i) the facets on the contacts of the rigid bodies are perpendicular to the vector connecting corresponding nuclei, $(x_{j}-x_{i})/l=n$; (ii) the volume is filled by bodies with no space left. Based on these two properties, the volume of the whole discretized specimen can be computed via summation over all contacts *e*

(1) $$V=\left\{\begin{array}{cc} \sum_{e}A^{e}l^{e}/2 & 2D\\ \sum_{e}A^{e}l^{e}/3 & 3D \end{array}\right.$$

As the system deforms, a displacement jump, **Δ**, occurs between bodies. The displacement jump is measured at the centroid of the contact facets, c. The contact strain in the normal direction, $e_{N}$, and tangential direction, $e_{T}$, are defined based on the projection of the displacement jump into the normal direction. The normal strain is represented by a real number and its direction is always n. In contrast, the tangential strain is represented by a vector perpendicular to n

(2) $$\begin{array}{c} e_{N}=\frac{n\cdot \Delta }{l}\;\;\;e_{T}=\frac{\Delta }{l}-e_{N}n \end{array}$$

Contact stresses are evaluated from contact strains

(3) $$\begin{array}{c} s_{N}=\left(1-D\right)E_{0}e_{N}\;\;\;s_{T}=\left(1-D\right)E_{0}\alpha e_{T} \end{array}$$

where $E_{0}$ and *α* are elastic parameters of the model dictating its elastic behavior. In the elastic regime, damage parameter *D* is null but may grow up to 1 in the inelastic regime. The evolution of the damage parameter is governed by two additional model parameters, meso-level tensile strength, $f_{t}$, and fracture energy in tension, $G_{t}$, and the straining direction *ω*

(4) $$\tan\omega =\frac{e_{N}}{\sqrt{\alpha }∥e_{T}∥}=\frac{\sqrt{\alpha }s_{N}}{∥s_{T}∥}$$

**Figure 3:**
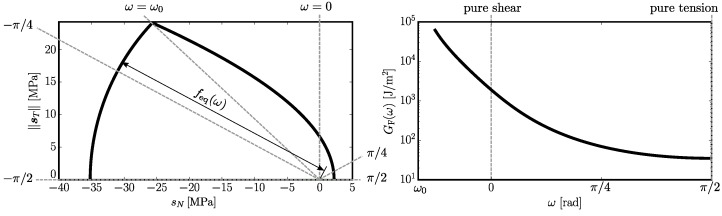
Constitutive relations for material parameters used in nonlinear models ($E_{0}=60\;$GPa, α=0.29, $f_{t}=2.2\;$MPa and $G_{t}=35\;$J/m${}^{2}$); length of the element is l=10mm. left: elastic envelope $f_{\mathrm{eq}}$ for different *ω*; right: fracture energy $G_{F}$ dependent on straining direction *ω*.

An element strained in direction *ω* behaves linearly until an equivalent stress $s_{\mathrm{eq}}=\sqrt{s_{N}^{2}+\alpha^{-1}{∥s_{T}∥}^{2}}$ reaches elastic limit $f_{\mathrm{eq}}$. The limit is dependent on *ω* and has two branches that intersects at $\omega_{0}$ (see Figure 3 left). The tensile part active in examples from Section 7 reads

(5) $$f_{\mathrm{eq}}\overset{\omega >\omega_{0}}{=}f_{t}\frac{4.52\sin\omega -\sqrt{20.0704\sin^{2}\omega +9\alpha \cos^{2}\omega }}{0.04\sin^{2}\omega -\alpha \cos^{2}\omega }$$

After reaching the strength, an exponential softening follows with total energy dissipation $G_{F}$. In pure tension (ω=π/2), the strength and fracture energy equal the model parameters $f_{t}$ and $G_{t}$. The more shear involved, the higher the strength and fracture energy. The fracture energy dependent on straining direction is plotted in Figure 3 on the right-hand side.

As stated previously, a complete description of the model’s kinematics (the relation between u, φ and **Δ**) and nonlinear constitutive equations can be found in [16]. The original, more complex and robust version of the nonlinear constitutive law is published in [18]. The simplification used here is obtained from the full model using the recommended values of the remaining model parameters. The nonlinear solution is found in incremental steps via an arc-length iterative solver; the model is static.

## 3. Simple Example

**Figure 4:**
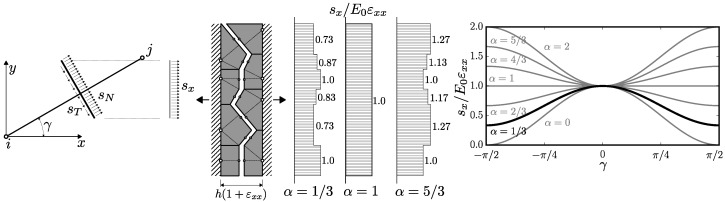
left: one element inclined by *γ* with normal and tangential stress projected into the *x*-direction; center: simple structure strained in the *x*-direction with normalized stress $s_{x}$ for three different *α* parameters; right: dependence of $s_{x}$ on *γ*—Equation (7).

Imagine a simple 2D setup of width *h* sketched in Figure 4. Eight bodies are strained in the *x*-direction by $\varepsilon_{xx}$ with restricted rotations and deformation in the *y*-direction. The displacement jump at all the contacts is clearly $\Delta =\left(h\varepsilon_{xx},\;0\right)$. Denoting γ∈〈0,π/2〉 is the angular deviation from the *x*-axis, the normal is $n=\left(\pm \cos\gamma ,\;\pm \sin\gamma \right)$ and length l=h/cosγ. The elastic normal and tangential stresses are (from Equations (2) and (3))

(6) $$s_{N}=E_{0}\varepsilon_{xx}\cos^{2}\gamma \;\;\;∥s_{T}∥=E_{0}\alpha \varepsilon_{xx}\sin\gamma \cos\gamma$$

and the stress acting in the *x*-direction on the projection of the facet to the *y*-direction is

(7) $$s_{x}=\frac{n\cdot \left(s_{N},\;∥s_{T}∥\right)}{\cos\gamma }=E_{0}\varepsilon_{xx}\left(\cos^{2}\gamma +\alpha \sin^{2}\gamma \right)$$

The stress $s_{x}$ transferred by the facet projections is shown in Figure 4 for three values of *α*. It increases with angular deviation *γ* for α>1 but decreases with *γ* for α<1. Therefore, the elements that are more inclined away from the straining direction behave in a stiffer or more compliant manner when *α* is larger or lower than 1, respectively. The inclined elements also have lower normal stress; shear stress becomes maximal when γ=±π/4.

This is the simple idea which lies behind this paper. In reality, there are also rotations and translations perpendicular to the stress direction. However, when the element is parallel to the straining direction, the active stress component is mainly normal stress. When inclined, shear stress is activated. Because the elements in the boundary layer are aligned with the boundary, the properties of the boundary layer differ. The same effect was recently used for generating anisotropic discrete model via introducing systematic bias into the angular distribution of the elements [23].

## 4. Macroscopic Elastic Behavior of a Discrete System

It is convenient to derive general elastic properties of a discrete system with unbiased geometry. This derivation has already been performed in 3D in Kuhl et al. [24]. The reader may wish to skip the following details regarding such derivation, moving straight to Equation (25), which summarizes the results of this section.

The analytical equations in the paper are hereinafter based on a simple assumption about the displacements and rotations of the bodies in the system when subjected to macroscopic uniform strain. It is assumed that all the rotations are null (φ=0), and the difference in displacements between any two nodes is

(8) $$u_{j}-u_{i}=\varepsilon \cdot \left(x_{j}-x_{i}\right)$$

The second order tensor ε is the applied uniform strain. This assumption is clearly not satisfied exactly, except in one special case when α=1. However, it will be shown that the real system elastic behavior is not very far from this assumption.

Using assumption (8) and perpendicularity of the facet area to the element, the displacement jump is determined by the strain tensor

(9) Δ=lε·n

The facet strains in the normal and tangential direction are (from Equations (2) and (9))

(10) $$\begin{array}{c} e_{N}=n\cdot \varepsilon \cdot n\;\;\;e_{T}=\varepsilon \cdot n-\left(n\cdot \varepsilon \cdot n\right)n \end{array}$$

According to Kuhl et al. [24], one can define the second order tensor N=n⊗n, the fourth order tensor $I=I_{ijkl}=\left(\delta_{ik}\delta_{jl}+\delta_{il}\delta_{jk}\right)/2$ with $\delta_{ij}$ being the Kronecker delta, and the third order tensor T=n·I−n⊗n⊗n. Then, Equation (10) can be rewritten as

(11) $$\begin{array}{c} e_{N}=N:\varepsilon \;\;\;e_{T}=T:\varepsilon \end{array}$$

The constitutive law in the elastic regime yields

(12) $$\begin{array}{c} s_{N}=E_{0}e_{N}=E_{0}N:\varepsilon \;\;\;s_{T}=E_{0}\alpha e_{T}=E_{0}\alpha T:\varepsilon \end{array}$$

The virtual work done by one element on virtual strain δε is

(13) $$\begin{array}{ccc} \delta W=Al\left(s_{N}\delta e_{N}+s_{T}\cdot \delta e_{T}\right) & = & AlE_{0}\left(\varepsilon :N⊗N:\delta \varepsilon +\alpha \varepsilon :T^{T}\cdot T:\delta \varepsilon \right)\\ & = & AlE_{0}\varepsilon :\left(N⊗N+\alpha T^{T}\cdot T\right):\delta \varepsilon \end{array}$$

To derive macroscopic elastic parameters, one starts with the equivalence of virtual work done by a mesoscopic discrete system and by macroscopic homogenized elastic continua [24]

(14) $$\delta W^{\mathrm{mes}}=\delta W^{\mathrm{mac}}$$

The virtual work of elastic continua in volume *V*, using σ=D:ε, is simply

(15) $$\delta W^{\mathrm{mac}}=V\sigma :\delta \varepsilon =V\varepsilon :D:\delta \varepsilon$$

while the work done by the discrete system is a summation of all contributions from individual contacts

(16) $$\delta W^{\mathrm{mes}}=\sum_{e}\delta W^{e}=\sum_{e}A^{e}l^{e}E_{0}^{e}\varepsilon :\left(N^{e}⊗N^{e}+\alpha T^{eT}\cdot T^{e}\right):\delta \varepsilon$$

The fourth order tensor of elastic constants follows on from Equations (15) and (16)

(17) $$D=\frac{1}{V}\sum_{e}A^{e}l^{e}E_{0}^{e}\left(N^{e}⊗N^{e}+\alpha T^{eT}\cdot T^{e}\right)$$

In the model used here, the normal stiffness, $E_{0}$, is identical for all the elements and can thus be moved outside the summation. Further simplification of this equation is possible, providing the model geometry is properly constructed. When there is no directional bias, the tensors N and T do not have any statistical dependence on elemental area, *A*, and length, *l*. Therefore, the summation can be rewritten using the mean value (E[−]) of terms with N and T

(18) $$D=\frac{E_{0}}{V}\left(E\left[N⊗N\right]+\alpha E\left[T^{T}\cdot T\right]\right)\sum_{c}A^{c}l^{c}$$

Both N and T are functions of normal n only; the normal should have the same probability of occurrence for any possible orientation. Using two independent random angles, *ξ* and *ζ*, with the following probability distribution function (pdf)

(19) $$f_{\xi }\left(\xi \right)=\left\{\begin{array}{cc} \frac{1}{2\pi } & \mathrm{for}\;\xi \in (0,2\pi )\\ 0 & \mathrm{otherwise} \end{array}\right.\;\;f_{\zeta }\left(\zeta \right)=\left\{\begin{array}{cc} \frac{\sin\zeta }{2} & \mathrm{for}\;\zeta \in (0,\pi )\\ 0 & \mathrm{otherwise} \end{array}\right.$$

the normal is $n=\left(\cos\xi ,\;\sin\xi \right)$ in 2D and n=(cosξsinζ,sinξsinζ,cosζ) in 3D. The mean values are calculated by analytical integration. Having arbitrary function G=g(ξ,ζ) dependent on two (or one in 2D) statistically independent random variables *ξ* and *ζ* with the probability distribution function $f_{\xi ,\zeta }\left(\xi ,\zeta \right)=f_{\xi }\left(\xi \right)f_{\zeta }\left(\zeta \right)$ (or $f_{\xi }\left(\xi \right)$ in 2D), the mean value of *G* is

(20) $$\mu_{G}=E\left(G\right)=\int_{-\infty }^{\infty }g\left(\xi \right)f_{\xi }\left(\xi \right)\;d\xi \;\;2D\;\;\;\mu_{G}=\int_{-\infty }^{\infty }\int_{-\infty }^{\infty }g\left(\xi ,\;\zeta \right)f_{\xi ,\zeta }\left(\xi ,\zeta \right)\;d\xi \;d\zeta \;\;3D$$

Evaluating Equation (20) for G=N⊗N and $G=T^{T}\cdot T$ yields

(21) $$\begin{array}{cc} E[N⊗N] & =\left\{\begin{array}{cc} \int_{0}^{2\pi }N⊗N\frac{1}{2\pi }\;d\xi =\frac{1}{4}\left(I+I^{\mathrm{vol}}\right) & 2D\\ \int_{0}^{2\pi }\int_{0}^{\pi }N⊗N\frac{\sin\zeta }{4\pi }\;d\zeta \;d\xi =\frac{2}{15}I+\frac{1}{5}I^{\mathrm{vol}} & 3D \end{array}\right. \end{array}$$

(22) $$\begin{array}{cc} E[T^{T}\cdot T] & =\left\{\begin{array}{cc} \int_{0}^{2\pi }T^{T}\cdot T\frac{1}{2\pi }\;d\xi =\frac{1}{4}\left(I-I^{\mathrm{vol}}\right) & 2D\\ \int_{0}^{2\pi }\int_{0}^{\pi }T^{T}\cdot T\frac{\sin\zeta }{4\pi }\;d\zeta \;d\xi =\frac{1}{5}\left(I-I^{\mathrm{vol}}\right) & 3D \end{array}\right. \end{array}$$

where $I^{\mathrm{vol}}=13\;1⊗1$ in 3D and $I^{\mathrm{vol}}=12\;1⊗1$ in 2D; 1 is the second order unit tensor.

Combining Equations (1), (18), (21) and (22), one obtains analytical formulas for the tensor of elastic constants

(23) $$D=\left\{\begin{array}{cc} E_{0}\left(\frac{1+\alpha }{2}I+\frac{1-\alpha }{2}I^{\mathrm{vol}}\right) & 2D\\ E_{0}\left(\frac{2+3\alpha }{5}I+\frac{3-3\alpha }{5}I^{\mathrm{vol}}\right) & 3D \end{array}\right.$$

For an isotropic elastic continuum, strain tensor D has the following form

(24) $$D=\left\{\begin{array}{cc} \frac{E}{1+\nu }I+\frac{2E\nu }{1-\nu^{2}}I^{\mathrm{vol}} & 2D,\mathrm{plane stress}\;\\ \frac{E}{1+\nu }I+\frac{2E\nu }{\left(1+\nu )(1-2\nu \right)}I^{\mathrm{vol}} & 2D,\mathrm{plain strain}\;\\ \frac{E}{1+\nu }I+\frac{3E\nu }{\left(1+\nu )(1-2\nu \right)}I^{\mathrm{vol}} & 3D \end{array}\right.$$

where *E* and *α* are Young’s modulus and Poisson’s ratio. Solving system of Equations (23) and (24), a set of equations relating elastic constants of homogenized continua, *E* and *ν*, and the discrete system, $E_{0}$ and *α*, are derived.

(25) $$\begin{array}{cccccccccccc} \alpha & =\frac{1-3\nu }{1+\nu } & E_{0} & =\frac{E}{1-\nu } & \Leftrightarrow & & \nu & =\frac{1-\alpha }{3+\alpha } & E & =E_{0}\frac{2+2\alpha }{3+\alpha } & & 2D,\mathrm{plane stress}\\ \alpha & =1-4\nu & E_{0} & =\frac{E}{\left(1-2\nu )(1+\nu \right)} & \Leftrightarrow & & \nu & =\frac{1-\alpha }{4} & E & =E_{0}\frac{\left(1+\alpha )(5-\alpha \right)}{8} & & 2D,\mathrm{plane strain}\\ \alpha & =\frac{1-4\nu }{1+\nu } & E_{0} & =\frac{E}{1-2\nu } & \Leftrightarrow & & \nu & =\frac{1-\alpha }{4+\alpha } & E & =E_{0}\frac{2+3\alpha }{4+\alpha } & & 3D \end{array}$$

### Numerical Verification

**Figure 5:**
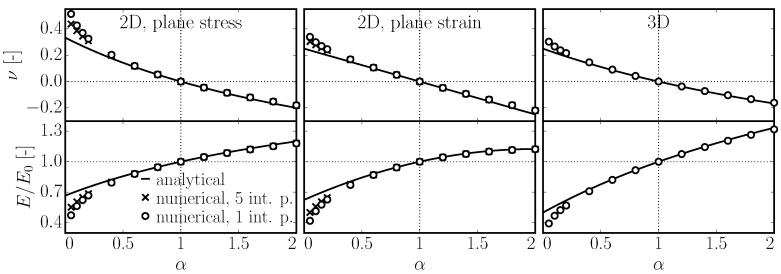
Comparison of macroscopic elastic modulus and Poisson’s ratio estimated analytically (Equation (25)) and computed numerically on a large discrete system.

The set of Equation (25) is numerically verified here. A large prism is loaded by stress $\sigma_{xx}$. The elastic parameters *E* and *ν* are found by fitting the displacements of the rigid bodies; see e.g. [16,25]. Figure 5 shows a comparison of the analytical formulas from Equation (25) and numerical results. An exact match only happens in case of α=1. Increasing or decreasing *α*, the discrete system yields a lower elastic modulus and higher Poisson’s ratio than values derived analytically. When *α* is close to zero, the system becomes unstable. Some improvement can be gained by calculating the elemental response not only at one point (centroid) but along the whole facet area, where the stresses generally differ due to the rotations of rigid bodies. The results with an improved 2D model that uses five points to integrate the stress on the facet are shown in Figure 5 as well. Since this improvement basically adds additional rotational stiffness to the elements, rigid body rotations are reduced and the system is stabilized. It shows slightly better agreement with analytical formulas.

## 5. Angular Bias in the Boundary Layer

The boundary layer shows strong bias in elemental orientation. In the ideal case when all the directions have the same probability, the angular deviation from the *x*-direction should have the following pdf

(26) $$\begin{array}{ccc} & 2D & 3D\\ f_{\gamma }\left(\gamma \right) & =\left\{\begin{array}{cc} 2/\pi & \mathrm{for}\;\gamma \in (0,\pi /2)\\ 0 & \mathrm{otherwise} \end{array}\right. & \;=\left\{\begin{array}{cc} \sin(\gamma ) & \mathrm{for}\;\gamma \in (0,\pi /2)\\ 0 & \mathrm{otherwise} \end{array}\right. \end{array}$$

The mean value (already defined in Equation (20)) and standard deviation of any function *G* dependent on *γ* are from the definition

(27) $$\mu_{G}=\int_{-\infty }^{\infty }g\left(\gamma \right)f_{\gamma }\left(\gamma \right)\;d\gamma \;\;\;\;\delta_{G}=\sqrt{\int_{-\infty }^{\infty }{\left(g\left(\gamma \right)-\mu_{G}\right)}^{2}f_{\gamma }\left(\gamma \right)\;d\gamma }$$

To calculate the mean and standard deviation of *γ*, Equation (27) is used with g(γ)=γ.

(28) $$\begin{array}{cc} \mu_{\gamma }=\left\{\begin{array}{cc} \frac{\pi }{4} & 2D\\ 1 & 3D \end{array}\right.\;\;\;\; & \delta_{\gamma }=\left\{\begin{array}{cc} \frac{\pi }{4\sqrt{3}} & 2D\\ \sqrt{\pi -3} & 3D \end{array}\right. \end{array}$$

**Figure 6:**
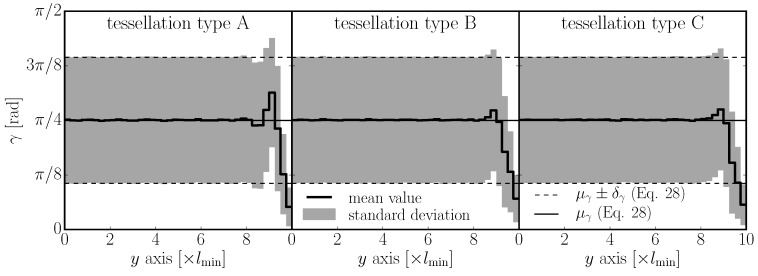
Statistical characteristics of angular deviation from the *x*-direction in 2D.

**Figure 7:**
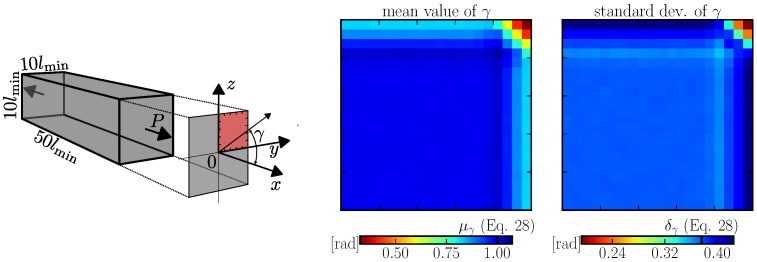
left: prism domain, angular deviation *γ* and emphasized quarter of the cross section; right: statistical characteristics of angular deviation from the *x*-direction in 3D.

These numbers are compared to the analysis of the actual discrete system in 2D (Figure 6) and 3D (Figure 7). In 2D, the discrete system was generated 500 times in a rectangular domain of size $20l_{\min}\times 100l_{\min}$, with a different seed of random generator each time. The weighted average and standard deviation of elemental inclination *γ* were calculated for different *y* locations; the weights were facet areas. Figure 6 shows one symmetrical half of the cross section in the range y=0 (center of the cross section) up to $y=10l_{\min}$ (boundary). The interior part is unbiased, while the boundary layer shows a strong decrease in the mean, indicating that the elements are more aligned with the boundary. Between the interior and the boundary, there is also a layer that exhibits a lower amount of aligned elements. All three tessellation types are investigated, and similar trends are observed.

In 3D, only tessellation type A is used. The discrete system was generated 3000 times in a prismatic domain $10l_{\min}\times 10l_{\min}\times 50l_{\min}$. The average and standard deviation of *γ* (weighted by facet area) are calculated for different locations in the cross section. Figure 7 shows a symmetrical quarter of the cross section ranging from y,z=0 (center of the cross section) up to $y,\;z=5l_{\min}$ (boundary). The bias in the boundary layer is similar to what was seen in 2D; it gets strongly emphasized in corners.

## 6. Effects on Elastic Behavior

The rectangle and prism from the previous section were loaded by straining in the *x*-direction ($\varepsilon_{xx}$), while deformations in the *y*- and *z*-direction were unconstrained. Such loading results in a single stress component $\sigma_{xx}$ and the following relations (these can be derived from Equation (24))

(29) $$\begin{array}{ccc} 3D\&2D\mathrm{plain stress} & \varepsilon_{xx}=\frac{\sigma_{xx}}{E} & \varepsilon_{yy}=\varepsilon_{zz}=-\frac{\nu \sigma_{xx}}{E}=-\nu \varepsilon_{xx}\\ 2D\mathrm{plain strain} & \varepsilon_{xx}=\frac{\sigma_{xx}\left(1-\nu^{2}\right)}{E} & \varepsilon_{yy}=-\frac{\sigma_{xx}\left(1+\nu \right)\nu }{E}=\frac{\nu }{\nu -1}\varepsilon_{xx} \end{array}$$

Since all the shear strains are zero and $\varepsilon_{yy}=\varepsilon_{zz}$, one can assume convenient rotation of the 3D coordinate system around the *x*-axis in such a manner that the elements are always parallel to plane xy and the normal $n=\left(\cos\gamma ,\;\sin\gamma \right)$ is the same for 2D and 3D models. The normal and tangential strain can be evaluated from Equation (11)

(30) $$\begin{array}{cc} e_{N} & =\varepsilon_{xx}\cos^{2}\gamma +\varepsilon_{yy}\sin^{2}\gamma \end{array}$$

(31) $$\begin{array}{cc} e_{T} & =\left(\cos\gamma \sin^{2}\gamma \left(\varepsilon_{xx}-\varepsilon_{yy}\right),\;\cos^{2}\gamma \sin\gamma \left(\varepsilon_{yy}-\varepsilon_{xx}\right)\right) \end{array}$$

and the magnitude of the shear strain is

(32) $$∥e_{T}∥=\sqrt{\cos^{2}\gamma \sin^{4}\gamma {\left(\varepsilon_{xx}-\varepsilon_{yy}\right)}^{2}+\cos^{4}\gamma \sin^{2}\gamma {\left(\varepsilon_{yy}-\varepsilon_{xx}\right)}^{2}}=∥\varepsilon_{xx}-\varepsilon_{yy}∥\sin\gamma \cos\gamma$$

Finally, strains $\varepsilon_{xx}$ and $\varepsilon_{yy}$ are substituted by Equation (29) and elastic constants *E* and *ν* by Equation (25). The stresses $s_{N}$ and $s_{T}$ are obtained by multiplying strains with $E_{0}$ and *α* according to Equation (12). All the three cases—3D, 2D plain strain and 2D plane stress—collapse into one

(33) $$\begin{array}{cc} s_{N} & =\varepsilon_{xx}E_{0}\left(\cos^{2}\gamma -\frac{1-\alpha }{3+\alpha }\sin^{2}\gamma \right) \end{array}$$

(34) $$\begin{array}{cc} ∥s_{T}∥ & =\varepsilon_{xx}E_{0}\alpha \sin\gamma \cos\gamma \frac{4}{3+\alpha } \end{array}$$

Assuming no directional bias, the mean value and standard deviations are calculated (Equation (27), $g\left(\gamma \right)=s_{N}$ or $g\left(\gamma \right)=∥s_{T}∥$)

(35) $$\begin{array}{cc} \mu_{s_{N}}=\left\{\begin{array}{cc} \varepsilon_{xx}E_{0}\frac{1+\alpha }{3+\alpha } & 2D\;\\ \varepsilon_{xx}E_{0}\frac{1+3\alpha }{3(3+\alpha )} & 3D \end{array}\right.\;\;\;\; & \delta_{s_{N}}=\left\{\begin{array}{cc} \varepsilon_{xx}E_{0}\frac{\sqrt{2}}{3+\alpha } & 2D\;\\ \varepsilon_{xx}E_{0}\frac{8}{3\sqrt{5}\left(3+\alpha \right)} & 3D \end{array}\right. \end{array}$$

(36) $$\begin{array}{cc} \mu_{∥s_{T}∥}=\left\{\begin{array}{cc} \varepsilon_{xx}E_{0}\alpha \frac{4}{\pi (3+\alpha )} & 2D\;\\ \varepsilon_{xx}E_{0}\alpha \frac{4}{3(3+\alpha )} & 3D \end{array}\right.\;\;\;\; & \delta_{∥s_{T}∥}=\left\{\begin{array}{cc} \varepsilon_{xx}E_{0}\alpha \frac{\sqrt{2(\pi^{2}-8)}}{\pi (3+\alpha )} & 2D\;\\ \varepsilon_{xx}E_{0}\alpha \frac{4}{3\sqrt{5}\left(3+\alpha \right)} & 3D \end{array}\right. \end{array}$$

These characteristics are based on (i) an ideal unbiased directional distribution of elements, therefore only the interior parts will behave accordingly; (ii) assumption (8), therefore these will be exactly satisfied only for α=1. For α≠1, the translations do not obey Equation (8) exactly and rotations are nonzero.

**Figure 8:**
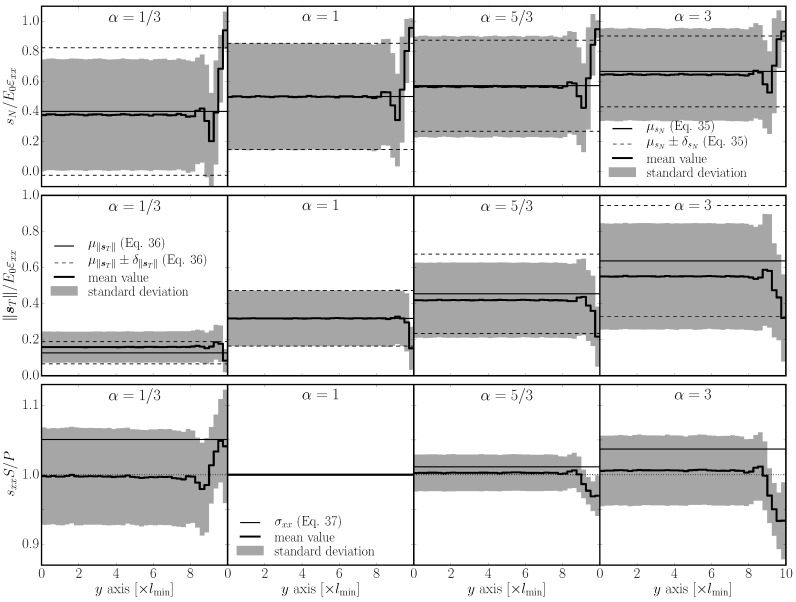
Average and standard deviation of normal stress ($s_{N}$, first row), shear stress ($s_{T}$, second row) and tensorial stress $s_{xx}$ (third row) calculated on a half cross section under tensile loading; 2D model.

**Figure 9:**
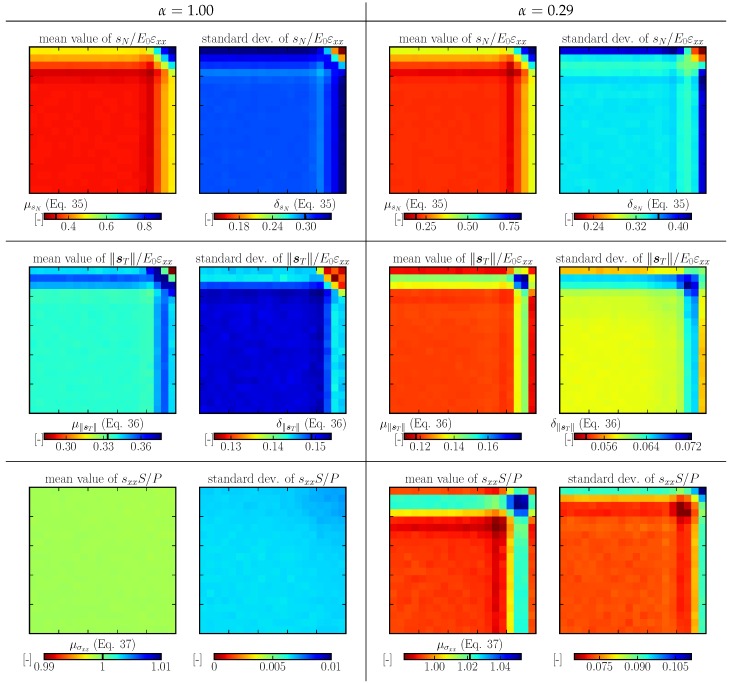
Average and standard deviation of normal stress, shear stress and tensorial stress; 3D model.

A comparison with real discrete system behavior is shown for 2D and 3D in Figure 8 and Figure 9 in the first two rows for selected *α* parameters. The value α=0.29 has been chosen for the 3D model because it is typical for concrete. The normal and shear stresses are normalized by $\varepsilon_{xx}E_{0}$; the calculation of the mean and standard deviation was weighted by facet area. Only tessellation type A is shown, as the other two types perform very similarly. One can see reasonable approximation of the stresses by analytical Equations (35) and (36) in the interior. Close to the boundary, the normal stress increases while the shear stress decreases. Moving further from the boundary, there is an opposite stress deviation as the amount of elements parallel with the boundary decreases.

Besides the stress at the contacts, one can also look at the average stress in the bodies. The tensorial stress, $\sigma_{xx}$, can be computed in three ways:

- from theoretical homogenized elastic continua, Equations (25) and (29)
  (37) $$\sigma_{xx}=E_{0}\varepsilon_{xx}\frac{2+2\alpha }{3+\alpha }\;\;2D\;\;\sigma_{xx}=E_{0}\varepsilon_{xx}\frac{2+3\alpha }{4+\alpha }\;\;3D$$
- from the total applied loading force, *P*, as P/S with *S* being the cross section area,
- in every rigid body using a fabric stress tensor with components $s_{ij}$
  (38) $$s_{ij}=\frac{1}{V_{0}}\sum_{e}c_{i}^{e}F_{j}^{e}\;\Rightarrow \;s_{xx}=\frac{1}{V_{0}}\sum_{e}c_{x}^{e}F_{x}^{e}$$
  where $V_{0}$ is the volume of the rigid body, and $c^{e}$ and $F^{e}$ are centroid and contact force of bond *e*; *e* runs over all contacts of one rigid body. An alternative approach for calculation of average tensorial stresses in the bodies [26] can be also employed with similar results.

The average and standard deviation (weighted by $V_{0}$) of tensorial stresses $s_{xx}$ are plotted in Figure 8 and Figure 9 in the third row. The stress is normalized by P/S and also compared to theoretical $\sigma_{xx}$. The boundary layer with a higher fraction of elements aligned with the straining direction is stiffer for a<1 and more compliant for a>1.

It is worth exploring whether α=1 is a sufficient condition to obtain elastically uniform structure. The contact force is $F=A\left(ns_{N}+s_{T}\right)=AE_{0}\left(ne_{N}+\alpha e_{T}\right)$ but reduces to $AE_{0}\Delta /l$ for α=1. If facets are perpendicular to elements and Equation (9) holds, then $F=AE_{0}\varepsilon \cdot n$. Starting with Equation (38) and assuming the same $E_{0}$ for all the elements, one arrives at

(39) $$s_{ij}=\frac{E_{0}}{V_{0}}\sum_{e}c_{i}^{e}A^{e}{\left(\varepsilon \cdot n^{e}\right)}_{j}=\frac{E_{0}}{V_{0}}\sum_{e}\sum_{k}c_{i}^{e}A^{e}\varepsilon_{jk}n_{k}^{e}=\frac{E_{0}}{V_{0}}\sum_{k}\varepsilon_{jk}\sum_{e}c_{i}^{e}A^{e}n_{k}^{e}$$

where the second and third expressions are just expanded multiplication and reordered summation. Finally, when all the facets of the rigid body forms an enclosed object, the last summation can be greatly simplified. Expression $\sum_{e}c_{i}^{e}A^{e}n_{k}^{e}$ is either 0 for i≠k or $V_{0}$ for i=k. Thanks to the symmetry of the strain tensor, one can write

(40) $$s_{ij}=E_{0}\varepsilon_{ij}$$

Three conditions were used to obtain the elastically uniform structure: (i) α=1; (ii) facets are perpendicular to elements; and (iii) the rigid bodies are enclosed. All three tessellation types from Section 2 meet these conditions.

## 7. Effects on Inelastic Behavior

The inelastic behavior of bonds is dependent on straining direction as well. A typical constitutive law is weaker and less ductile in tensile loading than in shear. Since the boundary layer contains more aligned elements, it becomes weaker and more brittle under straining along its direction than the interior part. Note that there are also nonlinear formulations independent from element orientation such as [27].

The results are reported only for the 3D model. The model parameters are taken from [16]: $E_{0}=60\;$GPa, α=0.29, $f_{t}=2.2\;$MPa and $G_{t}=35\;$J/m^2^. They were obtained by fitting a large experimental series [28].

### 7.1. Periodic Tension

**Figure 10:**
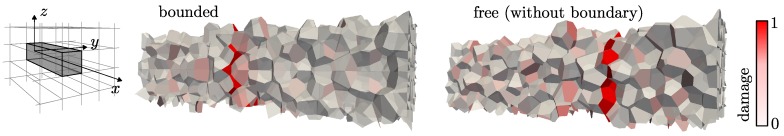
right: sketch of the periodic space; right: Damage in a 3D periodic model loaded in tension along the *x*-axis with and without a boundary.

In the first example, specimens with and without a boundary (tessellation type A) are compared. The removal of the boundary is achieved by periodic repetition of the model structure. Periodic prisms of 200 mm in length with square cross sections of 50 mm, 100 mm and 200 mm in size are loaded in tension; discretization size is $l_{\min}=10$ mm. The nominal stress and elongation of the prisms are calculated 30 times with different geometrical structures and averaged. Two variants are considered: (i) α=0.29 corresponding to typical concrete; and (ii) α=1.0 to show the behavior of an elastically uniform model in the nonlinear regime. Examples of damage patterns (localized into the macro-crack) in the specimen are shown in Figure 10. Note the mirrored structure in the central part of the bounded model.

**Figure 11:**
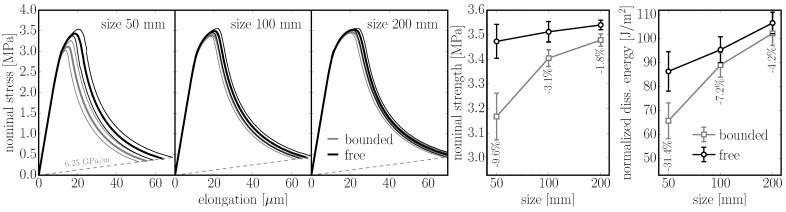
left: averaged responses of an elastically uniform periodic 3D model (α=1.0) loaded in pure tension. Models with boundaries (*bounded*) and without boundaries (*free*) of different size are compared; right: comparison of strength and dissipated energy.

**Figure 12:**
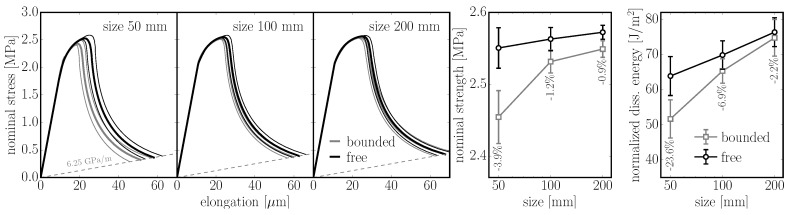
left: comparison of the averaged responses of a periodic 3D model of concrete with α=0.29 loaded in pure tension; right: comparison of strength and dissipated energy.

The results are shown in Figure 11 and Figure 12. One observes that both strength and dissipated energy grow with the size of the specimen. This phenomenon originates in the tortuosity of the crack; the crack path is more planar in smaller specimens simply because of the random nature of the geometry. The difference between the *bounded* and boundary *free* model decreases with size as the weak boundary layer occupies a lower portion of the specimen. In the worst case (the smallest specimen), the strength decreases by 10% (4%) and energy dissipation by 31% (24%) for α=1.0 (α=0.29, respectively) when boundaries are added.

### 7.2. Three-Point Bending

**Figure 13:**
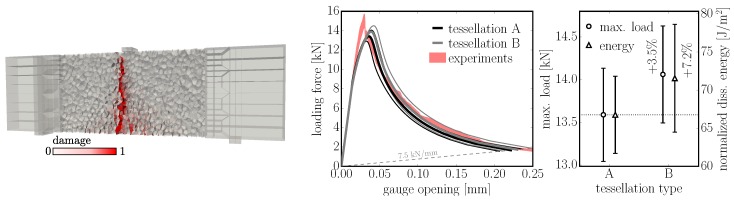
left: beam loaded in three-point bending; right: results averaged over 100 simulations.

The second example involves the three point bending of a concrete specimen. Two tessellation types, A and B, are compared on the second largest (n=2) unnotched (i=3) specimen tested in Grégoire et al. [28]. The depth of the beam is 200 mm, span is 500 mm and thickness is 50 mm, while the discretization size is $l_{\min}=10$ mm. The beam model with a localized macro-crack is shown in Figure 13, left. Investigated tessellation types A and B differ only in the initial placement of nuclei. Type A places nuclei only into the specimen domain while type B samples them into larger domains and then cuts those outside. One hundred simulations were computed for each case. The loading force and opening of the virtual gauge at the bottom surface were measured. A comparison of averaged responses, strengths and dissipated energies is shown in Figure 13, right. Tessellation type B is stronger by 4% and dissipates 7% more energy.

## 8. Conclusions

Discrete models with random geometry based on Voronoi and power tessellation were investigated under straining parallel with the boundary. It was found that, due to strong directional bias, the boundary layer has substantially different behavior than the interior part of the specimens. Analytical formulas for the interior part were derived and compared to the model behavior with reasonable agreement.

It was reported that stress in the boundary layer is transferred prevailingly by normal stress, while shear stress is reduced. Consequently, the boundary layer becomes stiffer for α>1 and more compliant for α<1. For a typical nonlinear constitutive law, the boundary layer has lower strength and lower ductility than the interior, irrespective of *α*.
